# Discrimination in the United States: Experiences of Native Americans

**DOI:** 10.1111/1475-6773.13224

**Published:** 2019-10-27

**Authors:** Mary G. Findling, Logan S. Casey, Stephanie A. Fryberg, Steven Hafner, Robert J. Blendon, John M. Benson, Justin M. Sayde, Carolyn Miller

**Affiliations:** ^1^ Department of Health Policy and Management Harvard T.H. Chan School of Public Health Boston Massachusetts; ^2^ Department of Psychology University of Michigan Ann Arbor Michigan; ^3^ Center for Human Identification University of North Texas Health Science Center Fort Worth Texas; ^4^ Research, Evaluation, and Learning Unit Robert Wood Johnson Foundation Princeton New Jersey

**Keywords:** American Indian and Alaska Natives, discrimination, Native Americans, racial/ethnic disparities in health and health care, racism, social determinants of health, survey research

## Abstract

**Objective:**

To examine reported racial discrimination and harassment against Native Americans, which broadly contribute to poor health outcomes.

**Data Source and Study Design:**

Data come from a nationally representative, probability‐based telephone survey including 342 Native American and 902 white US adults, conducted January‐April 2017.

**Methods:**

We calculated the percent of Native Americans reporting discrimination in several domains, including health care. We used logistic regression to compare the Native American‐white difference in odds of discrimination and conducted exploratory analyses among Native Americans only to examine variation by socioeconomic and geographic/neighborhood characteristics.

**Principal Findings:**

More than one in five Native Americans (23 percent) reported experiencing discrimination in clinical encounters, while 15 percent avoided seeking health care for themselves or family members due to anticipated discrimination. A notable share of Native Americans also reported they or family members have experienced violence (38 percent) or have been threatened or harassed (34 percent). In adjusted models, Native Americans had higher odds than whites of reporting discrimination across several domains, including health care and interactions with the police/courts. In exploratory analyses, the association between geographic/neighborhood characteristics and discrimination among Native Americans was mixed.

**Conclusions:**

Discrimination and harassment are widely reported by Native Americans across multiple domains of their lives, regardless of geographic or neighborhood context. Native Americans report major disparities compared to whites in fair treatment by institutions, particularly with health care and police/courts. Results suggest modern forms of discrimination and harassment against Native Americans are systemic and untreated problems.

## 1 INTRODUCTION

Native Americans have experienced worse health outcomes than whites since Europeans first arrived in the Americas more than 500 years ago.^1^ Centuries of massive trauma, genocide, forced migration, segregation, and discrimination have been important causes of Native Americans‐white health disparities, as well as poor health outcomes for generations of Native Americans.^1^, ^2^, ^3^, ^4^ However, because of sampling difficulties, Native Americans' modern experiences of discrimination and harassment remain understudied in health services research. In order to build evidence for appropriate policies and programs that address these problems and improve related health outcomes, it is critical to examine and document present‐day experiences of discrimination of Native Americans across a broad spectrum of life domains.

Previous research suggests that these experiences have had massive and cumulative effects on the physical, emotional, and psychological health of Native American individuals and communities.^1^, ^2^, ^3^, ^4^ Research also shows that experiences of discrimination and harassment (including disproportionate exposures to trauma and recurrent microaggressions) have severe negative consequences for Native Americans' health behaviors and related outcomes.^1^, ^2^, ^3^, ^4^, ^5^, ^6^, ^7^, ^8^, ^9^, ^10^, ^11^, ^12^ Major issues experienced by Native Americans include high mortality rates, poor health, low‐quality health care, suicide, drug and alcohol abuse, depression, and sexual violence.^5^, ^13^

Prior research indicates that for some US minorities, socioeconomic status, geographic variation, and neighborhood conditions may moderate the relationships between race, discrimination, and health. For example, discrimination research suggests that for blacks and Latinos, higher income and education levels are associated with greater reported discrimination.^14^, ^15^ However, it has not been thoroughly investigated whether these patterns would extend to Native Americans. Research also suggests that residential segregation impacts discriminatory experiences, with major consequences for racial/ethnic minorities' health, social mobility, and quality of life.^16^, ^17^, ^18^, ^19^, ^20^

Increasing evidence about the health risks associated with experiencing discrimination suggests an updated examination of minority groups is warranted, to complement ongoing national policy work on these issues.^20^, ^21^, ^22^, ^23^, ^24^ In particular, more research is needed among populations hard to reach in telephone polling, including Native Americans.^25^ Therefore, this study had three purposes: (a) to document the prevalence of racial discrimination against Native American adults across institutional domains (health care, education, employment, housing, political participation, police, and the criminal justice system), as well as interpersonal experiences that affect health outcomes, including slurs, microaggressions, harassment, and violence; (b) to document disparities in experiences by comparing Native Americans to whites; and (c) to conduct exploratory analyses examining the variation in Native American adults' experiences with discrimination by socioeconomic status and geographic/neighborhood characteristics.

This study brings a public health perspective to the complexity and pervasiveness of discrimination in the United States today, alongside complementary articles in this issue of *Health Services Research*. It was conducted as part of a larger nationally representative survey fielded in 2017 in response to a growing national debate about discrimination in the United States today,^22^, ^26^ to understand experiences of discrimination against several different groups in America, including blacks, Latinos, Asian Americans, Native Americans, women, and LGBTQ people.

## 2 METHODS

### 2.1 Study design and sample

Data were obtained from an original, nationally representative, probability‐based telephone (cell and landline) survey of US adults, conducted from January 26 to April 9, 2017. The survey was jointly designed by Harvard TH Chan School of Public Health, the Robert Wood Johnson Foundation, and National Public Radio. SSRS administered the survey. Because Harvard researchers were not directly involved in data collection and de‐identified datasets were used for analysis, the study was determined to be “not human subjects research” by the Harvard TH Chan School of Public Health Office of Human Research Administration.

The full sample included 3453 US adults aged 18 years and older, and this paper examines the subsample of 342 Native Americans and 902 non‐Hispanic whites. Potential respondents were told the surveyor was calling on behalf of the Harvard School of Public Health and National Public Radio, and the purpose of the survey was to conduct “research about some interesting issues in America today.” Screening questions regarding racial identities were asked at the beginning of the survey, and all questions about racial/ethnic identity were based on respondents' self‐identification. If respondents identified as multiracial, interviewers asked which race they identified with most. Respondents were asked if they identified as American Indian or Alaska Native, following the language used by the US Census, and volunteered responses of “Native American” are also allowed. In all follow‐up questions for Native American respondents, question wording used the term “Native American,” following language most commonly used. This method of screening also allowed interviewers to use the appropriate language in survey questions to describe or refer to the respondent's own identity. For example, this allowed questions to be read as “Did you experience [form of discrimination] because you are [‘Native American’]?” rather than “because of your race or ethnicity?”

The completion rate for this survey was 74 percent among respondents who answered initial demographic screening questions, with a 10 percent overall response rate, calculated based on the American Association for Public Opinion Research's (AAPOR) RR3 formula.^27^ Because data from this study were drawn from a probability sample and used the best available sampling and weighting practices in polling methods (eg, 68 percent of interviews were conducted by cell phone, and 32 percent were conducted via landline), they are expected to provide accurate results consistent with surveys with higher response rates^28^, ^29^ and are therefore reliably generalizable to the broader populations of white and Native American adults, within a margin of error of ±4.7 percentage points (whites) ±8.0 percentage points (Native Americans) at the 95 percent confidence interval. See Benson, Ben‐Porath, and Casey (2019) for a further description of the survey methodology.^30^

### 2.2 Survey instrument

The poll asked about adults' experiences of racial discrimination. We conceptualized racial discrimination as differential or unfair treatment of individuals based on self‐identified race, whether by individuals (based on beliefs, words, and behavior) or social institutions (based on laws, policies, institutions, and related behavior of individuals who work in or control these laws, policies, or institution).^15^, ^21^, ^31^ We analyzed 18 questions from the survey, covering six institutional and six interpersonal areas of discrimination (question wording in Appendix S1). Institutional areas included were health care, employment, education, housing, political participation, and police and courts. Interpersonal areas included were racial slurs, microaggressions, racial fear, sexual harassment, being threatened or nonsexually harassed, and violence. We also explored two areas in which concerns about discrimination might prevent adults from taking needed action: seeking health services and protection from the police.

Questions were only asked among a random half sample of respondents to maximize the number of questions while limiting respondent burden. Questions were only asked of relevant subgroups (eg, college questions only asked among adults who had ever applied to college). Questions on harassment, violence, and avoiding institutions for fear of discrimination were asked about whether they had been experienced by either respondents or their family members because of the sensitive nature of the topic. Prior literature has demonstrated the validity of asking questions this way to measure experiences on sensitive topics, as vicarious experiences of stress (eg, through discrimination or harassment experienced by family members) can adversely affect the health of individuals reporting it, even without respondents directly experiencing it themselves.^32^

### 2.3 Statistical analyses

After calculating descriptive statistics, we calculated the prevalence of all Native Americans and whites who reported that they had ever experienced racial discrimination in each of the domains. Using pairwise *t* tests of differences in proportions, we made uncontrolled comparisons of the percentage of Native American and white adults reporting discrimination across domains. For all analyses, statistical significance was determined at *P* < .05.

We then conducted logistic regression models to assess whether reporting discrimination remained significantly associated with race (reference group: whites) after controlling for the following variables that are related to variation in experiences of discrimination: gender, age (18‐49, 50+), household income (<$25 000, $25 000+), education (less than college degree or college graduate), neighborhood racial composition (whether or not respondents live in a neighborhood they describe as predominantly their own race), metropolitan status (urban, suburban, rural [outside metropolitan statistical areas]), and region (US Census Bureau 4‐region division: Midwest, Northeast, South, West).

Finally, we estimated logistic regression models as exploratory analyses among Native Americans only, to give further consideration as to whether socioeconomic status, neighborhood racial composition, residence on a reservation or tribal lands, or metropolitan status (rural or nonrural) are associated with experiences of institutional discrimination among Native American adults. We examined variation in institutional discrimination by socioeconomic status (income: <$25 000 or $25 000+; education: less than college degree or college graduate) and neighborhood racial composition (living in a predominantly Native American neighborhood or not), while controlling for gender and age (18‐49 or 50+). To test characteristics associated with experiencing greater amounts of discrimination across domains, we ran an ordinal logistic regression model reported in Table 3 to estimate factors associated with experiencing between 0 and 7 institutional types of discrimination among Native American adults only (questions were asked among only a half sample of respondents for each type of institutional discrimination). Logistic regression models were estimated using complete case analysis.

We tested the sensitivity of our results to several model specifications. First, we tested an alternate measure of neighborhood racial composition: whether respondents reported living on tribal lands such as a reservation, pueblo, or Alaska Native village (“yes” n = 109). Second, we tested an alternate measure of geography: whether respondents reported living in rural (n = 172) or nonrural (urban or suburban, n = 131) areas. We ultimately used living in a predominantly Native American neighborhood as the measure of neighborhood racial composition in final models, as it has been associated with worse health outcomes^19^, ^33^ and is more inclusive of Native American adults living off, but near, reservations and other tribal areas.

To compensate for known biases in telephone surveys (eg, nonresponse bias) and variations in probability of selection within and across households, sample data were weighted by household size and composition, cell phone/landline use, and demographics (gender, age, race/ethnicity, and Census region) to reflect the true population distribution of Native American and white adults in the country. According to the 2017 American Community Survey (ACS), Native American populations on average have slightly less phone access (95 percent) than the general population (97 percent). However, since access is still above 90 percent, we do not expect this slight difference to introduce any sample bias. ACS estimates were derived from data downloaded from the Integrated Public Use Microdata Series (IPUMS).^34^ Other techniques, including random‐digit dialing, replicate subsamples, and random selection of a respondent within a household, were used to ensure that the sample is representative. All analyses were conducted using STATA version 15.0 (StataCorp), and all tests accounted for the variance introduced by weighted data.

## 3 RESULTS

Weighted characteristics of Native Americans and whites in this study sample are presented in Table 1. Native Americans differed than whites on several demographic measures. Compared to whites, Native American adults were less likely to have a college degree (15 percent vs 34 percent, *P* < .01), more likely to live in lower‐income households (<$25 000/year) (39 percent vs 23 percent, *P* < .01), and less likely to live in a neighborhood that is predominantly their own race (31 percent vs 67 percent, *P* < .01).

**Table 1:** Characteristics of the study sample, by race^a^

	Native American (N = 342)^b^	White (N = 902)^b^	*P*‐value for difference^c^
	Percentage of respondents^d^		
Race^e^			
Native American or AI/AN only	73	—	—
Native American or AI/AN and White	23	—	—
Native American or AI/AN and Black	3	—	—
Native American or AI/AN and NHOPI	1	—	—
Ethnicity^e^			
Native American or AI/AN and Hispanic/Latino	8	—	—
Gender			
Male	50	48	.75
Female	50	52	.75
Age			
18‐49 y	56	48	.08
50+ y	44	52	.09
Education			
No college degree^f^	85	66	<.01*
College degree or more	15	34	<.01*
Household income			
<$25 000	39	23	<.01*
$25 000+	55	68	<.01*
Don't know/refused	7	9	.35
Enrolled in a tribe	49	—	—
Living on tribal lands^g^	23	—	—
Living in a neighborhood that is predominantly own race^h^	31	67	<.01*
Area of residence			
Urban	13	17	.24
Suburban	38	53	<.01*
Rural	45	25	<.01*
Don't know/refused	5	5	.81
US region of residence^i^			
Northeast	6	18	<.01*
Midwest	16	25	.02*
South	35	35	.99
West	39	18	<.01*
Don't know/refused	4	4	.84
Receives regular care from HIS/tribal/urban Indian clinics^j^	34	—	—

Abbreviations:  AI/AN, American Indian or Alaska Native; NHOPI, Native Hawaiian or Other Pacific Islander.

a Native American and non‐Hispanic white adults ages 18+.

b The sample size shown reflects the total number of respondents in each category.

c *P*‐value for difference is based on *t* tests.

d Percent of US population estimated with survey weights to adjust for unequal probability of sampling. Estimates may not add up to 100% due to rounding and don't know/refused responses that are included in the total n but not reported in this table.

e Some adults identified primarily as Native American or AI/AN, but also identified another racial or ethnic identity.

f Includes those with some college experience (including business, technical, or vocational school after high school) but no college degree, as well as those with a high school degree or GED certificate or less.

g Native American adults only asked whether they live on tribal lands such as a reservation, pueblo, or Alaska Native village.

h Question asked as: “People often describe some neighborhoods or areas as predominantly one group or another, such as a predominantly black or white neighborhood. Would you say that the area where you live is predominantly [Native American OR white], or not?”

i Regions defined by US Census Bureau 4‐region definition.

j Question asked as “Do you receive regular care from the Indian Health Service or tribal or urban Indian clinics?”

* indicates statistically significant difference between Native Americans and whites at *P* < .05.

Table 2 shows unadjusted estimates of Native American and white adults reporting personal discrimination because of their race across institutional and interpersonal domains, as well as actions based on concerns about discrimination. In the context of institutional discrimination, more than one in five Native American adults reported personally experiencing discrimination across most domains of life examined, including employment, health care, and the police and courts. For example, 33 percent of Native American adults said they experienced discrimination in obtaining equal pay or being considered for promotions, 31 percent in applying for jobs, 23 percent when going to a doctor or health clinic, and 29 percent when interacting with the police. Roughly one‐third reported discrimination against themselves or a Native American family member in being unfairly treated by the police (32 percent) and courts (32 percent). Less than one‐quarter of whites reported personally experiencing discrimination in any single domain.

**Table 2:** Differences between Native American and white adults in reporting discrimination because of race^a^

	Subject of discrimination^b^	N	Native American percent^c^	White percent^c^	*P*‐value for difference^d^
*Belief in overall discrimination*					
General belief that discrimination against [your race] exists today in the United States^e^	All adults	1244	75	55	<.01*
*Personal experiences of institutional discrimination*					
Employment					
Being paid equally or considered for promotions^f^	You	581	33	13	<.05*
Applying for jobs^g^	You	577	31	19	<.01*
Education					
Applying to or while attending college^h^	You	497	13	11	.61
Health care					
Going to a doctor or health clinic	You	646	23	5	<.01*
Housing					
Trying to rent a room/apartment or buy a house^i^	You	537	17	5	.02*
Political participation					
Trying to vote or participate in politics	You	598	10	4	.17
Police and courts					
Interacting with police	You	598	29	10	<.01*
Unfairly stopped or treated by the police^j^	You or family member	598	32	6	<.01*
Unfairly treated by the courts^j^	You or family member	598	32	7	<.01*
*Personal experiences of interpersonal discrimination*					
Microaggressions^k^	You	646	39	19	<.01*
Racial slurs^k^	You	646	35	23	.08
Racial fear^k^	You	646	10	7	.33
Violence^j^	You or family member	598	38	13	<.01*
Threatened or nonsexually harassed^j^	You or family member	598	34	16	<.01*
Sexual harassment^j^	You or family member	598	23	9	<.01*
*Actions based on concerns about discrimination*					
Avoided doctor or health care because of concerns of discrimination/poor treatment	You or family member	646	15	3	<.01*
Avoided calling the police because of concerns of discrimination	You or family member	598	22	2	<.01*

a Native American and non‐Hispanic white adults ages 18+. Individual questions only asked among a randomized subsample of half of respondents within each race category. Don't know/refused responses included in the total for unadjusted estimates.

b Questions about you are personal experiences only; questions about you or family member ask if items have happened to you or a family member because you or they are [Native American OR White]. All adults asked about discrimination against [Native Americans OR Whites] in America today.

c Percent calculated using survey weights.

d *P*‐value for difference between estimates using *t* tests.

e Question asked as “Generally speaking, do you believe there is or is not discrimination against [Native Americans OR whites] in America today?”

f Equal pay question only asked among respondents who have ever been employed for pay.

g Jobs question only asked among respondents who have ever applied for a job.

h College application/attendance was only asked among respondents who have ever applied for college or attended college for any amount of time.

i Housing question only asked among respondents who have ever tried to rent a room or apartment, or to apply for a mortgage or buy a home.

j Question wording: “Do you believe that you or someone in your family has [experienced/been _____] because you or they are [Native American OR white].”

k Question wording: “In your day‐to‐day life, have any of the following things ever happened to you, or not?” and respondent indicated they had experienced this *and* believed this happened because they are [Native American OR white]. Racial slurs = someone referred to you or a group you belong to using a slur or other negative word; microaggressions = someone made negative assumptions or insensitive or offensive comments about you; racial fear = people acted as if they were afraid of you.

* indicates statistically significant difference between Native Americans and whites at *P* < .05.

More than one‐third of Native American adults also reported that they have experienced several interpersonal forms of discrimination: 39 percent report that they have been the target of microaggressions, 38 percent said they or a family member have experienced violence because they are Native American, and 35 percent have been the target of racial slurs. Further, 34 percent say they or a family member have been threatened or nonsexually harassed because they are Native American, while 23 percent have experienced sexual harassment. Fewer reported that others have shown racial fear, that is, acted afraid of them because they are Native (10 percent).

Concerns or anticipation that they would experience discrimination also prevented some Native American adults from taking potentially needed actions: more than one in five (22 percent) reported that they have avoided calling the police or other authority figures, even when in need, and 15 percent reported that they have avoided the doctor or seeking health care for themselves or their family, out of fear they would be discriminated against or treated poorly.

**Figure 1:**
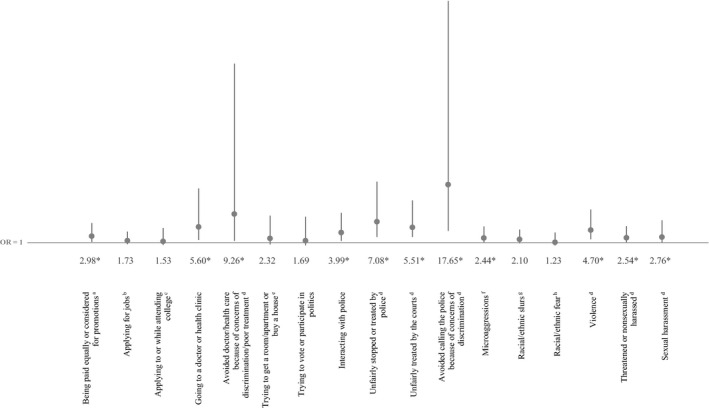
Adjusted odds of experiencing discrimination among Native American adults compared to whites (reference group). OR, odds ratio, with 95% confidence interval bars. Nationally representative sample of Native American and non‐Hispanic white adults ages 18+. Full model results available in Appendices S2 and S3. *Statistical significance at *P* < .05. Don't know/refused responses coded as missing. Odds ratios report the odds that Native American adults reported experiencing discrimination for each outcome (whites were the reference group). These estimates control for sex, age (18‐49 vs 50+), education (<college vs college graduate or more), household income (<$25 k vs $25 k+), living in a neighborhood that is predominantly one's own race, household location (urban, suburban, rural), and region (Northeast, Midwest, South, West). ^a^Equal pay question only asked among respondents who have ever been employed for pay. ^b^Jobs question only asked among respondents who have ever applied for a job. ^c^College application/attendance was only asked among respondents who have ever applied for college or attended college for any amount of time. ^d^Includes discrimination against you or a family member because you are Native American or white. ^e^Housing question only asked among respondents who have ever tried to rent a room or apartment, or to apply for a mortgage or buy a home. ^f^Microaggressions indicate that someone made negative assumptions or insensitive or offensive comments about you because you are Native American or white. ^g^Racial/ethnic slurs indicate that someone referred to you or your racial group using a slur or other negative word because you are Native American or white. ^h^Racial/ethnic fear indicates that people acted as if they were afraid of you because you are Native American or white

After we accounted for potential sociodemographic confounders in logistic regression models (Figure 1), Native American‐white disparities in reported discrimination persisted in most domains, including equal pay/promotions, health care visits, police interactions, unfair treatment of themselves or family members by the police and courts, microaggressions, violence, sexual and nonsexual threats/harassment toward themselves or family members, and avoiding health care and police protection due to anticipated discrimination against themselves or family members. Figure 1 shows adjusted differences in the odds of Native American adults personally experiencing discrimination compared to whites (full modeled results shown in Appendices S2 and S3).

Among Native Americans only, there were differences in odds of reporting discrimination by income and neighborhood racial composition, as shown in Table 3. Native Americans with household incomes of at least $25 000 annually had lower odds of experiencing discrimination in political participation and avoiding calling the police compared to those with less than $25 000 household incomes annually. For neighborhood racial composition, living in a predominantly Native neighborhood (a measure of residential segregation) was associated with higher odds of reporting discrimination in applying for jobs, equal pay/promotions, political participation, police interactions, and unfair treatment by the police and courts against you or Native American family members. In the ordinal logistic regression model, living in a predominantly Native neighborhood was the only variable associated with experiencing more types of discrimination overall (Table 3). College‐educated Native Americans had higher odds of reporting racial discrimination when trying to vote or participate in politics, while gender was not associated with discrimination for any domains in any model specifications.

**Table 3:** Odds of reporting personal experiences of racial discrimination across institutional domains among Native American adults in the United States^a^

	Employment		Health care		Housing	Political participation	Police and courts				Overall institutional discrimination
	Applying for jobs^c^	Equal pay/promotions^d^	Doctor or health clinic visits	Avoided doctor due to discrimination concerns	Trying to rent or buy a house^e^	Trying to vote or participate in politics	Interacting with police	Unfairly stopped or treated by the police	Unfairly treated by the courts	Avoided calling the police due to discrimination concerns	Discrimination across 0‐7 domains^f^
N^b^	152	156	144	146	101	155	156	163	164	164	310
**OR (95% CI)**											
Gender											
Male	Ref	Ref	Ref	Ref	Ref	Ref	Ref	Ref	Ref	Ref	Ref
Female	1.30 (0.45, 3.76)	1.96 (0.73, 5.25)	0.82 (0.27, 2.46)	1.09 (0.31, 3.82)	0.66 (0.19, 2.23)	0.43 (0.09, 1.99)	0.53 (0.18, 1.61)	0.62 (0.23, 1.69)	0.90 (0.35, 2.31)	0.62 (0.18, 2.21)	0.85 (0.43, 1.70)
Living in a predominantly Native American neighborhood											
No	Ref	Ref	Ref	Ref	Ref	Ref	Ref	Ref	Ref	Ref	Ref
Yes	5.07* (1.80, 14.21)	4.64* (1.78, 12.13)	2.08 (0.65, 6.68)	2.02 (0.59, 6.89)	1.60 (0.41, 6.17)	9.63* (2.26, 41.10)	6.94* (2.51, 19.14)	3.52* (1.37, 9.06)	3.47* (1.34, 8.96)	3.62 (1.03, 12.68)	3.03* (1.44, 6.39)
Education											
<College	Ref	Ref	Ref	Ref	Ref	Ref	Ref	Ref	Ref	Ref	Ref
College+	1.19 (0.37, 3.85)	1.45 (0.45, 4.65)	1.21 (0.37, 3.93)	1.43 (0.39, 5.29)	0.92 (0.24, 3.50)	4.70* (1.15, 19.16)	0.71 (0.22, 2.30)	0.66 (0.22, 1.95)	0.63 (0.21, 1.87)	1.26 (0.30, 5.28)	1.33 (0.64, 2.75)
Income											
<25 k	Ref	Ref	Ref	Ref	Ref	Ref	Ref	Ref	Ref	Ref	Ref
25 k+	0.57 (0.21, 1.54)	0.91 (0.35, 2.38)	0.43 (0.14, 1.32)	1.04 (0.33, 3.35)	0.77 (0.20, 2.89)	0.08* (0.02, 0.37)	0.63 (0.24, 1.66)	0.77 (0.29, 1.99)	0.65 (0.25, 1.65)	0.29* (0.10, 0.90)	0.57 (0.29, 1.13)
Age											
18‐49 y	Ref	Ref	Ref	Ref	Ref	Ref	Ref	Ref	Ref	Ref	Ref
50+ y	0.76 (0.28, 2.08)	1.01 (0.38, 2.68)	1.55 (0.52, 4.68)	0.86 (0.03, 2.38)	0.36 (0.09, 1.39)	1.44 (0.31, 6.70)	0.88 (0.33, 2.35)	0.76 (0.30, 1.93)	0.79 (0.31, 2.04)	0.47 (0.14, 1.54)	0.98 (0.52, 1.88)

Note

Nationally representative sample of Native American adults ages 18+.

Abbreviations: CI, confidence interval; OR, odds ratio.

a Too few cases of college attendance to include in models.

b Individual questions only asked among a randomized half sample of respondents. Don't know/refused responses coded as missing.

c Jobs question only asked among respondents who have ever applied for a job.

d Equal pay question only asked among respondents who have ever been employed for pay.

e Housing question only asked among respondents who have ever tried to rent a room or apartment, or to apply for a mortgage or buy a home.

f Ordinal logistic regression model with experiencing discrimination in 0‐7 institutional domains as the outcome; individual questions only asked among a randomized half sample of respondents, so the maximum number of times a respondent could report experiencing discrimination along any institutional questions was 7.

* Significant at *P* < .05.

In sensitivity analyses using alternate measures of geographic/neighborhood characteristics, living on tribal lands was associated with higher odds of reporting discrimination in obtaining housing (OR [95% CI] 6.15 [1.61, 23.52]) and unfair treatment of you or family members by the courts (3.13 [1.19, 8.24]); there was no association with living on tribal lands and overall institutional discrimination in ordinal logistic regression models (1.37 [0.67, 2.84]) or institutional discrimination in any other domains (data not shown). Using metropolitan status as a measure of geographic variation, compared to those living in rural areas, living in a suburban or urban area was associated with higher odds of reporting unfair treatment by the courts (OR [95% CI] 2.73 [1.02, 7.28]), but there was no association with rural status and reported overall institutional discrimination in ordinal logistic regression models (0.65 [0.34, 1.24]) or institutional discrimination in any other domains (data not shown).

## 4 DISCUSSION

Four key findings emerged from this survey of Native American adults. First, our results clearly demonstrate that Native Americans experience pervasive patterns of discrimination across many areas of life in the United States, particularly when it comes to health care, employment, interactions with the police and courts, and interpersonal areas including violence, harassment, microaggressions, and racial slurs.

Second, in the context of health care specifically, we found that almost one in six Native Americans reported avoiding health care for themselves or family members due to anticipated discrimination or unfair treatment. Prior research shows that Native Americans commonly report discrimination in health care visits, associate Western health care practices with other abuses by the US government, and deem such heath care as not culturally safe.^6^, ^35^ Our findings, coupled with prior research, demonstrate a need to improve accessible, affordable, and culturally appropriate care at both the institutional (eg, those serving Native American populations) and clinical levels (eg, by clinicians).

Third, Native Americans have significantly higher odds of reporting racial discrimination than whites in most areas, even after adjusting for major sociodemographic differences between the two groups. These significant differences in discrimination may amplify health disparities between Native Americans and whites.^1^, ^2^, ^3^, ^4^

Fourth, geographic/neighborhood measures indicated variation in discriminatory experiences. Native Americans who reported living in predominantly Native American areas had higher odds of reporting greater institutional discrimination overall, compared to those living in areas that were not predominantly Native American. This is generally consistent with related research showing that as the size of a racial/ethnic minority population increases, white attitudes become more biased against them—though importantly, results may vary by minority group and geographic characteristics.^36^, ^37^, ^38^ There may also be unmeasured geographic characteristics associated with living in self‐reported predominantly Native American neighborhoods that account for this relationship. Little research has examined discrimination among Native Americans and alternate measures of geography—whether living on tribal lands, or in rural areas—and our models showed no association with higher odds of reporting overall discrimination. For discrimination specifically by the court system, all three geographic measures found the same pattern: Whether by self‐reported rurality, living in a predominantly Native American area, or living on tribal lands, Native Americans in those areas had consistently higher odds of reporting unfair treatment by the courts (compared to, respectively, Native Americans in nonrural areas, not in predominantly Native areas, or off tribal lands). Due to our sample size, this study was limited in our ability to examine more nuanced patterns in experiences of discrimination along neighborhood and geographic lines, but future research should explore potentially important differences in Native Americans' experiences by geographic location, cultural identity, tribal affiliation, and residential segregation.^16^, ^17^, ^18^, ^19^, ^33^

Regardless of geographic or neighborhood living situation, Native American adults reported experiencing high levels of discrimination in many areas of life, and our estimates are consistent with nonrepresentative samples that also find discrimination and bias against Native American people in their interactions with the police and the courts.^39^, ^40^ This may be due in part to the complex criminal jurisdiction in Indian Country and also racial tension between Native and non‐Native communities, including suspicion of law enforcement, perceived prejudice, and cultural conflicts between Native American and Western values.^39^, ^40^, ^41^ This study found that more than one in five Native Americans reported avoiding interactions with the legal system because they fear unfair treatment, further perpetuating distrust and increasing racial disparities in interactions with law enforcement.

High levels of reported violence and harassment are also troubling, particularly given that such experiences are typically underreported in surveys,^42^ so the true rate is likely higher. These findings support other research documenting high rates of ongoing sexual assault and violence against Native American people, and Native American women in particular.^5^, ^13^ Results are also consistent with other research findings that Native Americans are frequently subject to recurrent microaggressions and racial slurs through antiquated and demeaning representations and stereotypes, including in sports mascots and media depictions.^4^, ^7^, ^8^ In addition to addressing more overt forms of discrimination, future programmatic and policy efforts should address these subtler, but still harmful forms of discrimination that threaten Native American identities.

Importantly, we found little variation in experiences of discrimination among Native American adults by socioeconomic status and gender, suggesting that having a higher income and earning a college degree are not protective against discrimination for Native Americans in most areas of life.

While it is beyond the scope of these results to recommend specific approaches to ending discrimination, because discrimination continues to affect such a significant share of the Native American population, health service researchers should continue to examine Native Americans' unique experiences of discrimination because of their long‐term impacts on patients' overall health and well‐being. Native Americans' problems with discrimination extend beyond health care, and future laws, policies, services, and research should identify, implement, and rigorously evaluate interventions to identify and end discrimination against Native Americans, as well as study‐related health and health care outcomes.

Taken together with other research documenting the failure of both federal policies and agencies to address the needs of Native nations,^23^, ^24^ this literature suggests that in addition to equalizing access to economic, medical, and social resources for Native Americans, specific antidiscriminatory efforts are necessary in future policies to improve health, including positive portrayals of contemporary Native Americans to change biased cultural ideas.^8^ Policies and programs that give Native nations better resources to address harassment and violence may also improve health for some, including the ability to exercise Special Domestic Violence Criminal Jurisdiction over non‐Indians under the Violence Against Women Act of 2013.^8^, ^43^

### 4.1 Limitations

The results of this study should be interpreted while considering the following limitations. Due to the cross‐sectional design of this study, we cannot determine the causality, timing, or severity of experiences of discrimination. Our low response rate is a notable limitation, though evidence suggests that low response rates do not bias results if the survey sample is representative of the study population.^28^, ^29^ Recent research has shown that such surveys, when based on probability samples and weighted using US Census parameters, yield accurate estimates in most cases when compared with both objective measures and higher response surveys.^28^, ^29^, ^44^, ^45^ For instance, a recent study showed that across fourteen different demographic and personal characteristics, the average difference between government estimates from high‐response rate surveys and a Pew Research Center poll with a response rate similar to this poll was 3 percentage points.^28^ However, it is still possible that some selection bias may remain that is related to the experiences being measured. This survey also did not distinguish between the heterogeneous experiences of different Native Americans, who are culturally diverse by language, heritage and traditions, geographic location, and tribal affiliation. The sample size also limited our ability to estimate complex models and to test geographic and neighborhood differences. Large confidence intervals in some logistic regression models (eg, health care avoidance) should be cautiously interpreted, as they may indicate low precision in estimates. In addition, because we specifically asked about racial discrimination, and because many forms of discrimination (including sexual harassment and violence) are often underreported, the “true” rate of Native Americans' experiences with discrimination is likely higher than our estimates. For example, in this issue SteelFisher et al^46^ explore the high rates of gender discrimination experienced among Native women. Given this, our findings may subject to underreporting and thus may be considered a lower bound estimate of discrimination and harassment against Native Americans in the United States today. Despite these limitations, our results highlight the extent of discrimination currently experienced by Native Americans across public policies and interpersonally.

## 5 CONCLUSIONS

Our findings document widespread, high levels of discrimination personally experienced by Native Americans today across many areas of life, regardless of geographic or neighborhood context. Alongside other research on the failure of federal policies and agencies to address the needs of Native communities, these results suggest discrimination against Native Americans is still a pervasive, systemic, and untreated problem in the United States. In policies, services, and research, future work should explicitly seek to end discrimination, as it affects a significant share of the Native American population.
