# Supplementary material for: Discrimination in the United States: Experiences of Native Americans
Source: Health Serv Res. 2019 Oct 27;54(Suppl 2):1431–41. doi: 10.1111/1475-6773.13224 (PMC6864378; doi:10.1111/1475-6773.13224)
Supplement: Supplementary file 2 [file HESR-54-1431-s002.docx]

**Appendix S1. Survey Questions for “Discrimination in the United States: Experiences of Native Americans”**

**Screening Questions on Racial/Ethnic Identity and Tribal Affiliation**

1. Are you, yourself, of Hispanic or Latino background, such as Mexican, Puerto Rican, Cuban, or other Latin American background?
2. (And besides being Latino if self-identified as Latino/Hispanic,) What race or races do you consider yourself to be? Please select one or more of the following categories.

White, Black or African American, Asian, American Indian or Alaska Native, Native Hawaiian or Other Pacific Islander, or Some other race (Specify) / (volunteered: don’t know/refused)

1. If respondent identified as more than one race/ethnicity: with which do you identify with more?

Hispanic or Latino, White, Black or African American, Asian, American Indian or Alaska Native, Native Hawaiian or Other Pacific Islander, or Some other race (Specify) / (volunteered: don’t know/refused)

1. Are you currently enrolled as a member with a Native American tribe?

**Screening Questions for Discrimination Questions**

1. Have you ever applied for a job?
2. (Half sample): Have you ever been employed for pay?
3. (Half sample): Have you ever applied for college or attended college for any amount of time?
4. (Half sample): Have you ever tried to rent a room or apartment, or to apply for a mortgage or buy a home?

**General perceptions of discrimination**

1. Generally speaking, do you believe there is or is not discrimination against [Native Americans OR Whites] in America today? Yes / No / (Volunteered response) Don’t know/Refused

**Institutional Discrimination**

1. (Ask if respondent has ever applied for a job): What about you? Do you believe you have ever personally experienced discrimination because you are [Native American OR White] **when applying for jobs?** Yes / No / (Volunteered response) Don’t know/Refused
2. (Ask if respondent has ever been employed): What about you? Do you believe you have ever personally experienced discrimination because you are [Native American OR White] **when it comes to being paid equally or considered for promotions**? Yes / No / (Volunteered response) Don’t know/Refused
3. What about you? Do you believe you have ever personally experienced discrimination because you are [Native American OR White] **when interacting with police**? Yes / No / (Volunteered response) Have never interacted with police/Don’t know/Refused
4. What about you? Do you believe you have ever personally experienced discrimination because you are [Native American OR White] **when trying to vote or participate in politics**? Yes / No / (Volunteered response) Have never tried to vote or participate in politics/Don’t know/Refused
5. What about you? Do you believe you have ever personally experienced discrimination because you are [Native American OR White] **when going to a doctor or health clinic**? Yes / No / (Volunteered response) Don’t know/Refused
6. (Ask if respondent has ever applied to or attended college): What about you? Do you believe you have ever personally experienced discrimination because you are [Native American OR White] **when applying to college or while at college**? Yes / No / (Volunteered response) Don’t know/Refused
7. (Ask if respondent has ever tried to rent/buy a place to live): What about you? Do you believe you have ever personally experienced discrimination because you are [Native American OR White] **when trying to rent a room or apartment or buy a house**? Yes / No / (Volunteered response) Don’t know/Refused

**Interpersonal Discrimination (Against You Only)**

(Rotate items B and C, always ask A last): In your day-to-day life, have any of the following things ever happened to you, or not?

How about (INSERT)?

(IF RESPONDENT ASKS WHAT ‘GROUP’ MEANS, PLEASE SAY: Such as your race, ethnicity, gender (or your sexual orientation or identity).)

Yes, has happened / No, has not happened / (Volunteered response) Don’t know/Refused

1. Someone referred to you or a group you belong to using a slur or other negative word
2. Someone made negative assumptions or insensitive or offensive comments about you
3. People acted as if they were afraid of you

(Q9-Q11) (If Yes to previous question): Do you believe this happened to you because of your race or ethnicity, your gender, (your sexual orientation or gender identity,) or was it for some other reason? You can select multiple answers.

Race or ethnicity, Gender, Sexual orientation, Gender identity, Or some other reason (SPECIFY)

**Interpersonal Discrimination (Against You or Family/Friend)**

(Scramble items A-E; always ask B right after A)

Do you believe that you or someone in your family has (INSERT ITEM) because you or they are [Native American OR White]? How about (INSERT ITEM)?

Yes / No / (Volunteered response) Don’t know/Refused

12. Experienced sexual harassment

13. Been threatened or non-sexually harassed

14. Been unfairly stopped or treated by the police

15. Been unfairly treated by the courts

16. Experienced violence

**Avoiding health care**

17. Have you ever avoided going to a doctor or seeking health care for you or others in your family out of concern that you would be discriminated against or treated poorly because you or they are [Native American OR White]? Yes / No / (Volunteered response) Don’t know/Refused

**Avoiding police/law enforcement**

18. Have you ever avoided calling the police or other authority figures, even when in need, out of concern that you or others in your family would be discriminated against because you or they are Native American? Yes / No / (Volunteered response) Don’t know/Refused

**Neighborhood Measures**

**Neighborhood racial composition**: People often describe some neighborhoods or areas as predominantly one group or another, such as a predominantly black or white neighborhood. Would you say that the area where you live is predominantly [Native American OR White], or not?

**Living on tribal lands:** Do you live on tribal lands such as a reservation, pueblo, or Alaska Native village? Yes / No / (Volunteered response) Don’t know/Refused

**Health Care:** Do you receive regular care from the Indian Health Service or tribal or urban Indian clinics?

**Appendix S2. Adjusted odds of experiencing discrimination among Native Americans compared to Whites (reference group) – Institutional Discrimination**

|  | **Equal pay/ promotions ^a^** | **Applying for jobs ^b^** | **Applying to or while attending college ^c^** | **Doctor or health clinic visits** | **Avoided doctor due to discrimination concerns ^d^** | **Trying to rent or buy a house ^e^** | **Trying to vote or participate in politics** | **Interacting with police** | **Unfairly stopped or treated by the police ^d^** | **Unfairly treated by the courts ^d^** | **Avoided calling the police due to discrimination concerns^d^** |
| --- | --- | --- | --- | --- | --- | --- | --- | --- | --- | --- | --- |
| **N** | 510 | 504 | 432 | 551 | 552 | 468 | 511 | 512 | 522 | 522 | 521 |
| OR (95% CI) | | | | | | | | | | | |
| **Race** |  |  |  |  |  |  |  |  |  |  |  |
| White | Ref | Ref | Ref | Ref | Ref | Ref | Ref | Ref | Ref | Ref | Ref |
| Native American | **2.98***  (1.32, 6.73) | 1.73  (0.70, 4.28) | 1.53  (0.45, 5.27) | **5.60***  (1.89, 16.57) | **9.26***  (1.65, 52.05) | 2.32  (0.61, 8.87) | 1.69  (0.34, 8.50) | **3.99***  (1.65, 9.64) | **7.08***  (2.71, 18.50) | **5.51***  (2.30, 13.15) | **17.65***  (4.48, 69.60) |
| **Gender** |  |  |  |  |  |  |  |  |  |  |  |
| Male | Ref | Ref | Ref | Ref | Ref | Ref | Ref | Ref | Ref | Ref | Ref |
| Female | 0.44  (0.17, 1.14) | 1.12  (0.50, 2.51) | 1.33  (0.46, 3.86) | 0.57  (0.18, 1.75) | **0.16***  (0.04, 0.68) | 0.37  (0.11, 1.22) | **0.25***  (0.07, 0.90) | 0.92  (0.37, 2.34) | 1.13  (0.40, 3.18) | 0.68  (0.22, 2.06) | 3.65  (0.97, 13.82) |
| **Living in predominantly own racial/ethnic identity neighborhood** | | | | |  |  |  |  |  |  |  |
| No | Ref | Ref | Ref | Ref | Ref | Ref | Ref | Ref | Ref | Ref | Ref |
| Yes | 0.61  (0.22, 1.71) | 0.53  (0.23, 1.26) | 1.02  (0.35, 2.97) | 0.63  (0.23, 1.72) | **7.26***  (1.28, 41.08) | 0.80  (0.18, 3.43) | 0.28  (0.07, 1.17) | 0.96  (0.33, 2.76) | 1.02  (0.31, 3.36) | 0.73  (0.25, 2.12) | 0.77  (0.14, 4.30) |
| **Education** |  |  |  |  |  |  |  |  |  |  |  |
| <College | Ref | Ref | Ref | Ref | Ref | Ref | Ref | Ref | Ref | Ref | Ref |
| College+ | **0.32***  (0.12, 0.81) | **0.24***  (0.09, 0.61) | 0.42  (0.12, 1.39) | 1.30  (0.47, 3.63) | **0.08***  (0.02, 0.35) | **0.23***  (0.08, 0.69) | **0.18***  (0.05, 0.73) | 0.38  (0.14, 1.04) | 0.50  (0.17, 1.44) | 0.40  (0.14, 1.09) | 0.44  (0.14, 1.43) |
| **Income** |  |  |  |  |  |  |  |  |  |  |  |
| <$25k | Ref | Ref | Ref | Ref | Ref | Ref | Ref | Ref | Ref | Ref | Ref |
| $25k+ | 0.47  (0.19, 1.14) | **2.81***  (1.02, 7.73) | 0.55  (0.19, 1.61) | **0.16***  (0.06, 0.45) | **0.08***  (0.02, 0.32) | 0.37  (0.09, 1.57) | **11.00***  (1.38, 87.63) | 0.95  (0.29, 3.07) | 1.22  (0.28, 5.28) | 1.55  (0.39, 6.22) | 0.83  (0.09, 7.41) |
| **Age** |  |  |  |  |  |  |  |  |  |  |  |
| 18-49 y | Ref | Ref | Ref | Ref | Ref | Ref | Ref | Ref | Ref | Ref | Ref |
| 50+y | 1.00  (0.34, 2.89) | 1.25  (0.55, 2.84) | 1.11  (0.38, 3.29) | **5.44***  (1.90, 15.59) | **5.47***  (1.16, 25.68) | 1.02  (0.26, 3.94) | 0.20  (0.06, 0.70) | 0.43  (0.15, 1.22) | 0.96  (0.27, 3.45) | 1.59  (0.48, 5.30) | 1.05  (0.16, 6.84) |
| **Area of residence** | | |  |  |  |  |  |  |  |  |  |
| Urban | Ref | Ref | Ref | Ref | Ref | Ref | Ref | Ref | Ref | Ref | Ref |
| Suburban | 0.58  (0.19, 1.72) | 0.80  (0.29, 2.23) | 0.34  (0.10, 1.17) | 0.63  (0.19, 2.06) | 0.44  (0.03, 6.38) | 0.83  (0.16, 4.41) | 0.33  (0.07, 1.56) | 0.98  (0.29, 3.32) | 0.80  (0.22, 2.91) | 0.95  (0.20, 4.44) | 0.44  (0.09, 2.17) |
| Rural | 0.47  (0.14, 1.58) | 0.44  (0.12, 1.55) | **0.13***  (0.02, 0.88) | 0.32  (0.07, 1.53) | 0.30  (0.02, 4.10) | 0.47  (0.05, 4.01) | 0.63  (0.12, 3.31) | 0.48  (0.12, 1.93) | 0.51  (0.11, 2.29) | 0.65  (0.16, 2.68) | **0.07***  (0.01, 0.35) |
| **Region of the country** | | |  |  |  |  |  |  |  |  |  |
| South | Ref | Ref | Ref | Ref | Ref | Ref | Ref | Ref | Ref | Ref | Ref |
| Northeast | 0.36  (0.08, 1.56) | 0.43  (0.14, 1.34) | 0.74  (0.17, 3.25) | 0.77  (0.19, 3.04) | 0.76  (0.09, 6.37) | 0.73  (0.11, 4.75) | 2.91  (0.36, 23.62) | 1.40  (0.36, 5.41) | 2.91  (0.57, 14.76) | 0.54  (0.08, 3.68) | 8.61  (0.77, 95.94) |
| Midwest | 1.35  (0.42, 4.37) | 0.36  (0.12, 1.08) | 1.51  (0.35, 6.57) | 0.73  (0.16, 3.26) | **0.03***  (0.01, 0.15) | 0.31  (0.05, 2.12) | **11.90***  (1.55, 91.68) | 1.08  (0.32, 3.60) | 1.59  (0.35, 7.27) | 1.01  (0.26, 3.89) | **8.73***  (1.16, 60.54) |
| West | 0.62  (0.18, 2.06) | **0.28***  (0.10, 0.79) | 1.02  (0.24, 4.35) | 0.89  (0.26, 3.11) | 1.06  (0.23, 4.83) | 1.13  (0.25, 5.13) | **8.12***  (1.38, 47.72) | 0.52  (0.10, 2.72) | 2.02  (0.42, 9.64) | 0.95  (0.24, 3.69) | 9.63  (0.88, 104.85) |

Table notes: OR=Odds Ratio, CI=95% Confidence Interval. Nationally representative sample of Native American and non-Hispanic White adults ages 18+. *Indicates statistical significance at p<0.05. Don’t know/refused responses coded as missing. Odds ratios report the odds that Native American adults reported experiencing discrimination for each outcome (Whites were the reference group). These estimates control for gender, age (18-49 vs. 50+), education (<college vs. college graduate or more), household income (<$25k vs. $25k+), living in a neighborhood that is predominantly one’s own race, household location (urban, suburban, rural), and region (Northeast, Midwest, South, West). ^a^ Equal pay question only asked among respondents who have ever been employed for pay. ^b^ Jobs question only asked among respondents who have ever applied for a job. ^c^ College application/attendance was only asked among respondents who have ever applied for college or attended college for any amount of time. ^d^ Includes discrimination against you or a family member because you are Native American or White. ^e^ Housing question only asked among respondents who have ever tried to rent a room or apartment, or to apply for a mortgage or buy a home.

**Appendix S3. Adjusted odds of experiencing discrimination among Native Americans compared to Whites (reference group) – Interpersonal Discrimination**

|  | **Microaggressions ^a^** | **Racial/ethnic slurs ^b^** | **Racial/ethnic fear ^c^** | **Violence ^d^** | **Threatened or non-sexually harassed ^d^** | **Sexual harassment ^d^** |
| --- | --- | --- | --- | --- | --- | --- |
| **N** | 553 | 553 | 552 | 522 | 521 | 520 |
| OR (95% CI) | | | | | | |
| **Race** |  |  |  |  |  |  |
| White | Ref | Ref | Ref | Ref | Ref | Ref |
| Native American | **2.44***  (1.04, 5.72) | 2.10  (0.91, 4.84) | 1.23  (0.37, 4.04) | **4.70***  (2.09, 10.56) | **2.54***  (1.11, 5.82) | **2.76***  (1.01, 7.51) |
| **Gender** |  |  |  |  |  |  |
| Male | Ref | Ref | Ref | Ref | Ref | Ref |
| Female | **0.38***  (0.19, 0.76) | 0.63  (0.31, 1.26) | **0.26***  (0.08, 0.86) | 0.47  (0.21, 1.06) | **0.39***  (0.17, 0.89) | 0.85  (0.34, 2.14) |
| **Living in predominantly own racial/ethnic identity neighborhood** | | | | |  |  |
| No | Ref | Ref | Ref | Ref | Ref | Ref |
| Yes | 0.60  (0.29, 1.24) | 0.73  (0.36, 1.51) | 3.28  (0.75, 14.33) | 0.67  (0.26, 1.68) | 0.51  (0.21, 1.23) | 0.63  (0.22, 1.84) |
| **Education** |  |  |  |  |  |  |
| <College | Ref | Ref | Ref | Ref | Ref | Ref |
| College+ | 1.02  (0.51, 2.01) | 0.51  (0.26, 1.00) | 0.43  (0.15, 1.24) | 0.61  (0.25, 1.50) | 0.47  (0.19, 1.13) | 0.60  (0.19, 1.91) |
| **Income** |  |  |  |  |  |  |
| <$25k | Ref | Ref | Ref | Ref | Ref | Ref |
| $25k+ | 0.75  (0.32, 1.76) | 1.87  (0.71, 4.94) | 0.41  (0.12, 1.35) | 0.97  (0.35, 2.65) | 0.98  (0.38, 2.53) | 1.88  (0.62, 5.70) |
| **Age** |  |  |  |  |  |  |
| 18-49 y | Ref | Ref | Ref | Ref | Ref | Ref |
| 50+y | **0.49***  (0.26, 0.96) | **0.42***  (0.21, 0.84) | 0.45  (0.16, 1.30) | 0.53  (0.22, 1.27) | 0.55  (0.24, 1.25) | 0.61  (0.21, 1.74) |
| **Area of residence** | | |  |  |  |  |
| Urban | Ref | Ref | Ref | Ref | Ref | Ref |
| Suburban | 1.25  (0.53, 2.94) | 1.13  (0.49, 2.63) | 0.92  (0.21, 3.98) | 2.31  (0.81, 6.61) | 0.79  (0.28, 2.21) | 3.24  (0.81, 13.03) |
| Rural | 0.90  (0.29, 2.78) | 0.51  (0.18, 1.47) | 0.94  (0.22, 4.04) | 0.56  (0.15, 2.03) | **0.21***  (0.06, 0.78) | 2.01  (0.42, 9.57) |
| **Region of the country** | | |  |  |  |  |
| South | Ref | Ref | Ref | Ref | Ref | Ref |
| Northeast | 1.68  (0.67, 4.20) | **2.49***  (1.04, 5.97) | 0.22  (0.04, 1.26) | 0.89  (0.21, 3.83) | 1.07  (0.29, 3.91) | 0.80  (0.18, 3.65) |
| Midwest | 0.66  (0.27, 1.63) | 1.06  (0.41, 2.73) | 0.55  (0.14, 2.22) | 1.40  (0.50, 3.93) | 1.22  (0.42, 3.56) | 0.44  (0.13, 1.47) |
| West | 1.02  (0.40, 2.61) | 0.87  (0.35, 2.16) | 1.19  (0.26, 5.34) | 1.49  (0.46, 4.83) | 1.55  (0.53, 4.50) | 0.74  (0.18, 3.06) |

Table notes: OR=Odds Ratio, CI=95% Confidence Interval. Nationally representative sample of Native American and non-Hispanic White adults ages 18+. *Indicates statistical significance at p<0.05. Don’t know/refused responses coded as missing. Odds ratios report the odds that Native American adults reported experiencing discrimination for each outcome (Whites were the reference group). These estimates control for gender, age (18-49 vs. 50+), education (<college vs. college graduate or more), household income (<$25k vs. $25k+), living in a neighborhood that is predominantly one’s own race, household location (urban, suburban, rural), and region (Northeast, Midwest, South, West). ^a^ Microaggressions indicate that someone made negative assumptions or insensitive or offensive comments about you because you are Native American or White. ^b^ Racial/ethnic slurs indicate that someone referred to you or your racial group using a slur or other negative word because you are Native American or White. ^c^ Racial/ethnic fear indicates that people acted as if they were afraid of you because you are Native American or White. ^d^ Includes discrimination against you or a family member because you are Native American or White.
